# Cohabitation and Marriage Formation in Times of Fertility Decline: The Case of Sweden in the Twenty-First Century

**DOI:** 10.1007/s10680-024-09703-9

**Published:** 2024-05-22

**Authors:** Stefano Cantalini, Sofi Ohlsson-Wijk, Gunnar Andersson

**Affiliations:** Department of Sociology, Stockholm University Demography Unit (SUDA), Stockholm University, 106 91 Stockholm, Sweden

**Keywords:** Union formation, Cohabitation, Marriage, Sweden

## Abstract

Developments over time in the prevalence of marriage and cohabitation formation has long received much interest, but less is known about more recent developments for different population subgroups in European countries. This applies as well to Sweden, a country considered a forerunner in family-demographic change. In contrast, much attention has been paid to the falling birth rates during the 2010s, and explanations that focus on the role of increasing uncertainties. In the Swedish case, the fertility decline has been documented across all main socio-demographic subgroups. The objective of this study is to examine whether the same situation holds for first marriage and cohabitation formation during the 2010s and the exceptional years of the Covid-19 pandemic. Based on Swedish population registers, including with new cohabitation data, we present annual indices of first marriage formation (1991–2022) and cohabitation formation (2012–2022) across a number of socio-demographic strata. We demonstrate a continuous decline in first marriage formation since the early 2010s with an additional sharp dip during the pandemic and a post-pandemic recovery. In contrast, there was a remarkable stability in cohabitation formation during 2012–2022. Although socio-demographic groups differ in their overall levels of marriage and cohabitation formation, the recent trends are strikingly similar across groups. Cohabiting couples, across population subgroups, have become less inclined to transition their union status to a more committed level, as manifested by marriage or parenthood. This occurred in spite of a positive economic climate in the 2010s and stable family policies, indicating that other forces are at play.

## Introduction

Trends in union formation have undergone several well-documented changes in developed societies since an often used historical benchmark of the 1950s. These changes have been theoretically debated in relation to family change in a broader sense, and Sweden has often been seen as a forerunner in such change, not least regarding the general decline in marriage (Goldscheider et al., 2015; Lesthaeghe, 2010; Ohlsson-Wijk et al., 2020). Since the 1960s, Sweden was among the first countries to experience a steady decrease in marriage rates, which continued into the 1990s (Andersson, 1998; Andersson & Kolk, 2015). This long-term decline in marriage propensities was coupled with a gradual increase in the prevalence of cohabitation (Heuveline & Timberlake, 2004). However, it was subsequently followed by a marriage-trend reversal in the opposite direction during the early 2000s (Ohlsson-Wijk, 2011). For the next decade, however, there is limited information about union formation in Sweden, as the searchlight among family demographers has mainly been directed towards the structure of the fertility declines that occurred during the 2010s (Ohlsson-Wijk & Andersson, 2022). These declines, which also comprised its Nordic neighbours (Comolli et al., 2021; Hellstrand et al., 2021), occurred “unexpectedly”, despite a policy setting that still supports a sound work–family balance and continuously high levels of gender equity within couples—factors previously connected to non-depressed fertility levels (Esping-Andersen, 2009; McDonald, 2000).

Thus, the question is whether the decade-long decline in fertility—which scholars have attributed to increasing perceptions of uncertainty about the future (Guetto et al., 2021; Neyer et al., 2022; Vignoli et al., 2020)—also means less family and intimate relationships in other dimensions, for example in terms of less cohabitations and marriages. Recent studies reveal that the 2010s fertility fall in Sweden (Neyer et al., 2022)—as well as in Finland (Hellstrand et al., 2022)—was connected to lower marriage rates, but was not due to declining union formation overall. Rather it was mainly driven by declining fertility within cohabiting unions. In this study, we aim at looking closer into this issue, by investigating the most recent trends in marriage and cohabitation formation in Sweden. We thus ask if the formation of first marriages and cohabitations has decreased in a similar manner as first-birth rates, which have been the main driver of the fertility decline (Ohlsson-Wijk & Andersson, 2022) in the 2010s, and we follow the same trends through the Covid-19 pandemic in 2020–2021 and the post-pandemic year 2022. Moreover, we aim at studying whether recent trends and trend changes have depended on changes in the socio-demographic composition of men and women during the 2010s, as defined by birth parity, labour market status and migration background, or are characterized by different behaviours across subgroups of the population thus investigating possible inequalities in union formation. In this respect, previous studies on fertility change during the same period showed that the declining trends in first-birth rates were largely consistent across social groups (Ohlsson-Wijk & Andersson, 2022). In this study, we ask whether similar conclusions can be drawn for patterns in cohabitation and marriage formation.

With this work, we extend the previous literature in three directions. First, we update the trends of first marriage formation in Sweden up to 2022, focusing on both women and men, thus providing new evidence on marriage propensities in the context of recent fertility decline and during the Covid-19 pandemic and its immediate aftermath. (The most recent study on marriage formation trends in Sweden covered years up to 2012 and focused only on the female population; cf. Andersson & Kolk, 2015.) Second, we provide insight into the possible relationship between trends in union formation and childbearing during the 2010s. For instance, changes in marriage formation can be affected by fertility changes (Ohlsson-Wijk, 2011). In this study, we investigate this issue by studying the extent to which trends in marriage formation can be explained by changes in the parity composition of women and men at family formation ages and if they differ across parity groups. Finally, for the first time in Sweden, we are able to rely on administrative register data to study total population trends in cohabitation formation. Sweden has previously lacked all-encompassing data of such nature. This is of general interest as the country is known as a forerunner in the normalization of cohabitation (Heuveline & Timberlake, 2004) and as marriage and childbearing in this context are almost always preceded by cohabitation (Andersson et al., 2017).

## Background

### Marriage Trends in Sweden

From the 1960s to the 1990s, Sweden experienced a steady decline in marriage rates, which primarily involved childless and never-married women (Andersson, 1998). This decline can be explained by a general decrease in the popularity of marriage as the only institution for family life, in favour of unmarried cohabitation, which was already prevalent in Sweden in the early 1970s and which increased further in the next decades (Heuveline & Timberlake, 2004). Moreover, the decline in marriage rates was driven by a general postponement of the age at marriage, parallel to the postponement of childbearing. Taken together, the increase of informal unions and the postponement of marriage and childbearing paved the way for a typical pattern of family formation that was characterized by entry into cohabitation and followed by the birth of children and, in some cases, by marriage (Andersson, 1998; Holland, 2013). In fact, slightly more than half of all children and two-thirds of first-born children are currently born to unmarried parents (Thomson, 2023), suggesting that marriage is no longer seen as a prerequisite for childbearing. However, there is still a connection between the two events, as marriage is strongly linked to childbearing and often considered a way of formalizing a commitment that has already been strengthened by having children (Duvander, 1999; Lappegård & Noack, 2015; Moors & Bernhardt, 2009).

The secular decline in marriage rates in Sweden had only two deviations, presumably linked to changes in legislation. A first small increase in marriage rates occurred during 1974–1976, as a result of the liberalization of divorce laws, which made divorce quicker and easier to obtain. These legislative changes, which also included a simplification of the procedures for marriage formation, led a body of newly divorced individuals to re-enter the marriage market and may also have increased the popularity of marriage as a more modern contemporary institution (Andersson, 1998; Agell, 1982). A second large peak in marriage rates occurred in 1989, in response to the near abolition of the widows’ pensions for women that were not married as of January 1990. A large number of cohabiting couples thus married in 1989 as they perceived that it could secure a better future pension in the event of a partner’s death (Hoem, 1991).

After a long period of marriage decline, only temporarily halted in 1974 and 1989, Sweden experienced a marriage “revival” in the 2000s (Ohlsson-Wijk, 2011). A substantial peak in marriages occurred in 2000, perhaps because of some digital preferences of people to marry in that year (Ohlsson-Wijk, 2014), which was followed by a slight dip in 2001, but after that the long-term downward trend in marriage rates was replaced by a continuous and steady upward trend over the first decade of the 2000s (Andersson & Kolk, 2015). This increase can be explained by four factors as spelled out by Ohlsson-Wijk (2011). First, it may partly be attributed to a “recuperation” of postponed marriages at higher ages. Second, part of the reversal was explained by changes in the composition of the population in terms of increasing proportions of individuals with a solid labour market attachment as well as by increasing fertility trends during the same decade—factors that positively affect the propensity to marry in Sweden. Third, it is possible that a higher prevalence of cohabiting unions in those years could have stimulated marriage rates through a larger number of couples who eventually transformed their informal union into marriage. Finally, in contrast to what happened in the previous decades, the popularity of marriage seemed to have increased in the first years of the 2000s.

Theoretically, the long-term decrease in marriage rates could be interpreted within the framework of the second demographic transition. According to this framework, a shift towards “post-materialist” values related to secularization, individualization and autonomy increasingly affects family dynamics (Lesthaeghe & van de Kaa, 1986; van de Kaa, 2002). This approach seemed to fit family changes in Sweden in the second half of the 1900s better than other concurrent ideas. For instance, studies found little support for Becker’s argument (1991) according to which the decrease in the popularity of marriage was related to women’s higher labour market participation and related economic independence (e.g. Andersson, 2000). Oppenheimer’s (1994) proposition that the fall in marriage rates in the USA could be linked to young men’s deteriorating labour market conditions did also not apply to Sweden, since the labour market prospects for Swedish men did not worsen dramatically until the 1990s (Andersson, 2000).

More recently, theorists of gender equality and the gender revolution have argued that the decline in family formation, including marriage, during the second half of the 1900s may be due to a decreasing willingness to commit to a partner. This is primarily seen as driven by the growing burden on women, as they increasingly took part in the labour market and at the same time still carried the main responsibility for housework and childcare, but also as driven by unclear norms and expectations regarding the roles within couples (Goldscheider et al., 2015). Accordingly, the willingness to commit to a partner will increase if men’s involvement in the family sphere increases, potentially reversing any declining trends of marriage formation. This perspective could potentially be applied to explaining not only the Swedish marriage decline from the 1960s onwards, but also the Swedish marriage “reversal” in the early 2000s, when the country in many respects had experienced growing gender equality in the family—especially compared to other developed societies (Esping-Andersen & Billari, 2015; Goldscheider et al., 2015).

### Marriage and Cohabitation in Sweden

Before 2011, unmarried cohabitation could be detected in Swedish population-register data only if a couple had a common child through which the two partners could be linked to a common residential property, making it difficult to study patterns in non-marital cohabitation at the population level. However, studies based on survey data have provided valuable insights into cohabitation in Sweden. For instance, unmarried cohabitation was already prevalent in the 1970s and is nowadays widely considered a common precursor to marriage, making it rare to marry without cohabiting first (Andersson & Philipov, 2002; Heuveline & Timberlake, 2004). This is reflected in statistics on family formation based on survey data that show that at age 35, for the period 2007–2013, 89–91% of Swedes had started a first union as a cohabitation and only 3% as a marriage; the remaining fraction had not entered a coresidential union at all (Andersson et al., 2017). Moreover, the median age at first union formation was 23–24 years, whereas the median age at first marriage formation was 35–37 years (ibid.), suggesting that marriage is almost always preceded by relatively long periods of cohabitation (Holland, 2013). In general, however, the majority of Swedes eventually still marry sometime during their lives (Ohlsson-Wijk, 2011). For example, in the period 2007–2013, around 70% of them had formed a marriage before age 50 (Andersson et al., 2017).

In addition, legal and institutional differences between cohabitation and marriage in Sweden are very limited, with similar rights for cohabitors and married couples in many policy areas (Perelli-Harris & Sánchez Gassen, 2012). For instance, the provision of different welfare-state schemes is often targeted at the individual rather than the (married) couple and, in case of parental union dissolution, both married and cohabiting parents are granted the same rights of child custody (Schiratzki, 2008). However, some differences between the two forms of union still exist in terms of inheritance in the event of a partner’s death and in relation to pensions, making marriage slightly more beneficial than cohabitation from a long-term financial perspective (Perelli-Harris & Sánchez Gassen, 2012).

Considering the large diffusion of cohabitations and small legal differences towards marriage, some scholars have argued that the decision among Swedish couples to marry is mainly driven by practical and pragmatic reasons (Heuveline & Timberlake, 2004), whereas others argue that marriage primarily has a symbolic meaning, for example as offering a more definite and committed relationship (Ohlsson-Wijk et al., 2022; Perelli-Harris et al., 2014). In this respect, studies have indicated that cohabitation may emerge as a particularly preferred family form in times of increasing economic and social uncertainties (Perelli-Harris et al., 2014), as in the aftermath of the Great Recession and during the Covid-19 pandemic (Guetto et al., 2021). It has been argued that increasing uncertainties associated with prior experiences (referred to as “shadows of the past”) as well as future prospects (“shadows of the future”; cf. Bernardi et al., 2019) have contributed to the fertility declines in the 2010s (Comolli et al., 2021). The same factors may also have driven couples to choose cohabitation over marriage due to its lower level of commitment.

## Data and Methods

### Data, Study Population and Events

Our analyses are based on Swedish population and administrative registers, gathered and organized at Statistics Sweden. Swedish registers allow us to reconstruct longitudinal information on family-demographic histories and socio-economic information for the full resident population. In our work, we also take advantage of a new register on apartments and households, enabling us for the first time to study cohabitation trends in Sweden at the population level.

We analyse two main family formation events, namely the risk of first marriage formation and the risk of entry into cohabitation. Swedish registers offer complete marriage histories for the population since 1968, allowing us to distinguish between first and subsequent marriages. Conversely, household data are available only from 2011,^1^ thus lacking information on cohabitation histories prior to that year, and not specifying cohabitation order. To address this limitation, we focus our cohabitation analyses on a relatively young section of the population (ages 18–28), assuming that the first observed cohabitation in the data also represents individuals’ first cohabitation experience (see below).

We only consider opposite-sex family formation. The gender-equal opportunity for same-sex marriage only became available in 2009 (Andersson & Noack, 2010), and non-marital cohabitation can only be measured for opposite-sex couples. As for the first marriage risk, we restrict our analysis to never-married Swedish-born men and women aged 18 to 55 during 1991–2022, focusing on cohorts born between 1946 and 2004. Age 18 is set as the lowest age since this is the minimum legal age for marriage in Sweden. In order to put the marriage trends during the 2010s in context, we also include years from 1991 in our analysis, covering both the previous period of marriage decline in the 1990s and the period of marriage revival in the first decade of the 2000s (Andersson & Kolk, 2015; Ohlsson-Wijk, 2011). The total study population includes 2,196,117 never-married women and 2,448,468 never-married men, observed during 32,364,963 and 38,210,891 person-months of non-married exposure, respectively.

For cohabitation formation, we exploit the definition of a cohabiting couple given by Statistics Sweden. According to this definition, persons in a cohabiting couple are either persons in an unmarried relationship who have a common child and are registered in the same dwelling (apartment or single-family house); or unmarried persons who do *not* have a common child and are registered in the same dwelling, but in the latter case only if (i) both are aged 18+, (ii) they are of opposite sex, (iii) the age difference between them is < 15 years and (iv) they are not relatives. In this case, only one cohabiting couple can be identified within the same household. Therefore, in our study the transition into the status of a cohabiting person according to this definition is considered as an entry into cohabitation. We limit our analysis to never-married Swedish-born men and women who were not cohabiting in 2011, or who turned 18 later than 2011. We follow their risk of cohabitation formation from 2012 to 2022. We are mainly interested in the formation of a first coresidential union, and since the data contain no information on cohabitation histories prior to 2011, we focus our attention on the cohorts born from 1983 to 2004, being aged 18 to 28 during 2012–2022.^2^ The total study population includes 803,375 non-cohabiting women and 920,076 non-cohabiting men, observed during 3,659,578 and 4,633,968 person-years of non-cohabiting exposure, respectively.

### Variables

The main independent variable of our study is *calendar year*, represented by single years of exposure from 1991 to 2022 for the analysis of marriage formation and from 2012 to 2022 for the analysis of cohabitation formation. A set of covariates representing various demographic and socio-economic control variables are also included in the models. *Age*—entered as yearly dummies—refers to the age at the end of each calendar year. *Parity* is a time-varying variable that is divided into four categories: no children, one child, two children and three or more children. *Region of residence* allows us to control for differences in union formation in different types of settlement (see Duvander, 1999 regarding marriage). The 290 Swedish municipalities are divided into three broad categories: large cities and the commuting municipalities near those cities (Stockholm, Gothenburg, Malmö); medium-sized towns and municipalities near those towns (commuting and non-commuting alike); and small towns, commuting municipalities near small towns, and rural areas. *Parents’ country of birth* is a time-constant variable divided into four categories: both parents born in Sweden; at least one Nordic-born parent (i.e. both Nordic-born or one Nordic and one Swedish-born); at least one non-Nordic European-born parent (i.e. both non-Nordic European or one non-Nordic European and one Swedish/Nordic-born); and at least one non-European parent (i.e. both non-European or one non-European and one Swedish/Nordic/European-born).^3^*Educational attainment* is a time-varying variable consisting of four categories: primary; secondary or tertiary less than 2 years; tertiary 2–3 years; and tertiary four or more years. Finally, *labour market activity* is an eight-category time-varying variable based on work-related earnings before tax and unemployment and student benefits in the previous year (see Ohlsson-Wijk & Andersson, 2022). Earnings cut-off points for five equally sized categories were determined based on the 2011 income distribution among Swedish men and women aged 18–65. These five categories (i.e. quintiles) range from a lowest annual earnings level of 36,600 SEK^4^ (€3,357 in late 2022 exchange rates) and are adjusted for inflation annually. The lower bound for each quintile is Q1: SEK36,600 (€3,357), Q2: SEK169,300 (€15,528), Q3: SEK260,400 (€23,884), Q4: SEK318,800 (€29,241), Q5: SEK399,500 (€36,642). The earnings quintiles are not sex-specific, allowing for objective comparisons over time and between the sexes. Three additional categories include the unemployed (receiving unemployment benefits and earning less than the second earnings quantile), students (receiving student allowances and earning less than the second earnings quintile) and inactive individuals (earning less than the base level and not categorized as unemployed or a student).

### Methods and Analytical Strategy

For our analyses, we rely on event-history techniques, in the form of piece-wise constant baseline intensity models, estimated separately for women and men. Entry into first marriage is analysed with the accuracy of monthly precision. Time at risk for marriage formation starts at the month an individual turns 18 and ends at the month of any first marriage, first emigration, death, turning age 55 or December 2022, whichever occurs first. Entry into cohabitation is analysed with the accuracy of yearly measurement, since monthly information on informal unions are not available in our register data.^5^ In this case, time at risk starts the year a person turns 18 or 2012, whichever occurs last, and ends the year of entering cohabitation, first emigration, entering marriage, death, turning age 28 or 2022, whichever occurs first.

We proceed with our analytical strategy in two steps. The first part focuses on the main effects in marriage and cohabitation trends and investigates whether they are driven by compositional changes related to childbearing and socio-economic factors. We estimate two models for each event of interest. The first standardizes the union formation trends—i.e. the association between calendar year and marriage/cohabitation formation—for the effect of age only (Model 1), whereas the second adds variables related to parity, region of residence, parents’ country of birth, educational attainment and labour market activity (Model 2).

We present the main trends in union formation by providing time series of relative risks of the propensity to form a first marriage or cohabiting union by each calendar year under risk, relative to a suitable baseline year (for previous examples of this approach in relation to marriage formation, see Andersson, 1998; Ohlsson-Wijk, 2011; Andersson & Kolk, 2015).

In the second step of our analytical strategy, we focus on socio-demographic (in)equalities in the trends of union formation, analysing whether the main trends vary between individuals with different characteristics. To this end, our Model 2 is estimated with an interaction between calendar year and each additional variable of interest (cf. Andersson, 1998; Andersson & Kolk, 2015; Ohlsson-Wijk & Andersson, 2022), namely parity, parents’ country of birth and labour market activity. To not overly burden the text, we do not present results from models interacting calendar year with region of residence and education, respectively. However, additional analyses (available upon request) show results for these variables that provide similar information as that provided for the factors we include in our presentation.

## Empirical Results

### First Marriage Trends

Before displaying the trends in marriage formation, Table 1 presents the relative risks of marriage formation across the various levels of parity, region of residence, parents’ country of birth, educational attainment and labour market activity, as estimated in our Model 2, without interactions and separately for women and men. The propensity to marry is substantially lower for childless individuals than for parents. More specifically, childless women and men have a marriage risk that is 48% (${RR}_{parity0}=$ 0.52) and 56% (${RR}_{parity0}=$ 0.44) lower than for parents of one child, respectively. Moreover, marriage propensities increase slightly with the number of children, especially for women, with mothers of two and three or more children being 21% and 31% more likely to marry than mothers of one child. Further, those living in small towns have slightly lower marriage formation risks than those living in medium-sized towns and, especially, large cities. Moreover, men and women with Nordic and European-born parents have similar marriage risks to individuals with two Swedish-born parents, whereas those—especially women—with non-European parents are more likely to enter marriage than others. Finally, there is a clear positive educational and labour market attachment gradient, which looks similar as previously found for first-birth risks (Andersson, 2000; Ohlsson-Wijk & Andersson, 2022), with less advantaged men and women (e.g. low educated, unemployed, inactive and low-income earners) displaying lower marriage intensities than individuals with higher educational attainment or earnings.

**Table 1:** Relative risk of first marriage formation, by parity, region of residence, parents’ country of birth, education and labour market activity. Piece-wise constant baseline intensity models for women and men separately

	Women	Men
*Parity*		
0	0.52	0.44
1	1	1
2	1.21	1.11
3+	1.31	1.19
*Region*		
Large cities	1	1
Medium-sized towns	0.93	0.91
Small towns	0.87	0.83
*Parents’ country of birth*		
Swedish-born	1	1
Nordic-born	0.99	0.94
Eur-born	1.07	1.03
Non-Eur-born	1.35	1.17
*Education*		
Primary	1	1
Secondary or tertiary < 2 years	1.24	1.25
Tertiary 2–3 years	1.82	1.75
Tertiary 4+ years	2.38	2.18
*Labour market activity*		
Inactive	0.71	0.63
Student	0.73	0.86
Unemployed	0.90	0.83
Low income (Q1)	1	1
Medium–low income (Q2)	1.08	1.21
Medium income (Q3)	1.21	1.39
Medium–high income (Q4)	1.35	1.58
High income (Q5)	1.55	1.89

Models also control for calendar year and age. Q1 = First quintile; Q2 = Second quintile; Q3 = Third quintile; Q4 = Fourth quintile; Q5 = Fifth quintile

*Source*: Swedish register data, authors’ own calculations

**Fig. 1:**
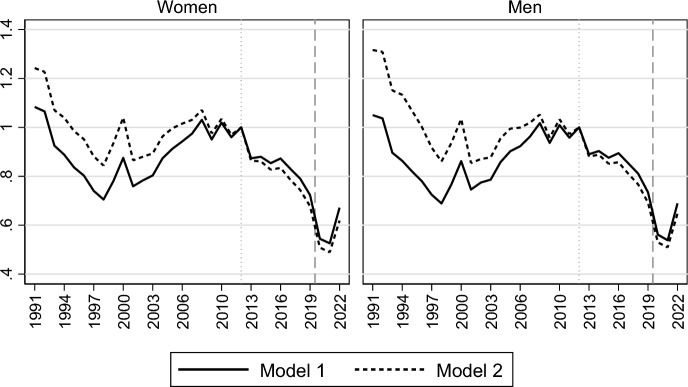
Relative risk of first marriage formation in Sweden, by calendar year. Piece-wise constant baseline intensity models for women and men separately. *Notes*: Rates relative to rates in 2012. Model 1 controls only for age; Model 2 also controls for parity, region of residence, parents’ country of birth, educational attainment and labour market activity

Turning to the period trends in marriage formation, Fig. 1 presents how the marriage intensities evolved for Swedish women and men during 1991–2022, with marriage formation risks being presented relative to those in 2012 as the baseline year, and with the onset of the Covid-19 pandemic being marked with a dashed vertical line in our diagram. The solid lines present results from the model that standardizes the trends for only age (Model 1), which show a slow but gradually accelerating decline in first marriage rates during the 2010s, followed by a sharp drop during the pandemic and a subsequent recovery in its immediate aftermath in 2022. The marriage risk declined by almost 30% from 2012 to 2019 (${RR}_{women2019}=$ 0.72; ${RR}_{men2019}=$ 0.73) and by an additional dramatic 24–25% in the first year of the pandemic (${RR}_{women2020}=$ 0.54; ${RR}_{men2020}=$ 0.56).^6^ The marriage formation risk remained at a depressed level in the second year of the pandemic, in 2021. With the end of the pandemic, marriage rates recovered in 2022, although not fully returning to their pre-pandemic levels. The trends during 2010–2019 resemble those previously found for first births (Neyer et al., 2022; Ohlsson-Wijk & Andersson, 2022), but the drastic decline in marriage formation during the Covid-19 pandemic is specific for that life-course transition.

The dashed lines—corresponding to a model that includes controls for changes in parity, parents’ country of birth, region of residence, educational attainment and labour market activity (Model 2)—appear very similar to the solid ones, suggesting that the recent decline in first marriage rates was not driven by compositional changes related to socio-demographic factors or by the parallel fertility decline and its related changes in the parity distribution of the never-married population. The small discrepancy between the two lines mainly depends on the inclusion of the variable on labour market activity in the model, which contributes to making the marriage decline in the last decades even more visible than without that control. The proportions of women and men usually more prone to enter marriage—i.e. those active in the labour market and with high earnings (see Table 1)—increased during the 2010s (Ohlsson-Wijk & Andersson, 2022). Thus, the improving economic situation during this time period seems to partially counteract the decline in marriage propensities. On the contrary, the inclusion of the variable for parity—which controls for the increasing proportion of childless individuals over the last decade—makes the marriage decline somewhat less steep, but the differences between models with and without this control are negligible (results available on request).

**Fig. 2:**
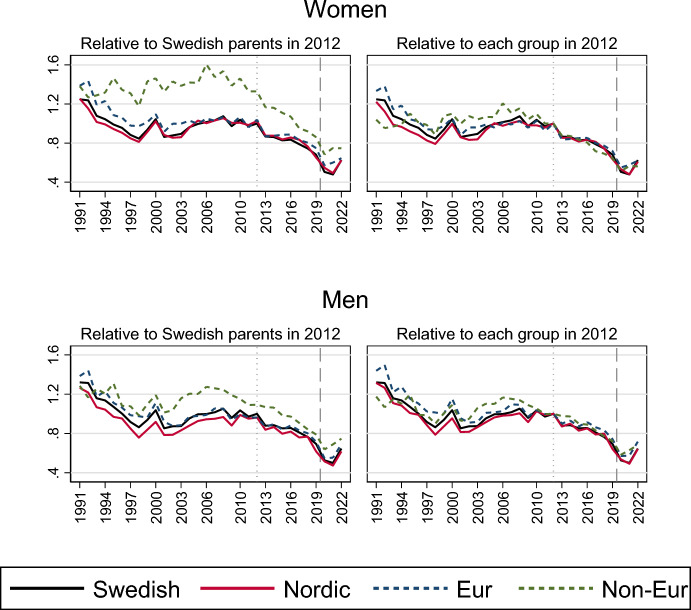
Relative risk of first marriage formation in Sweden, by calendar year and parents’ country of birth. Piece-wise constant baseline intensity models for women and men separately. *Notes*: The left panel shows rates relative to those with Swedish-born parents in 2012; the right panel shows rates relative to those in 2012 for each category of parents’ birth country. Models control for age, parity, region of residence, education and labour market activity

Turning to the role of any inequalities in marriage formation trends, Fig. 2 shows how trends differ according to the birth country of the never-married persons’ parents. The left panel presents the marriage risks relative to individuals with Swedish-born parents in 2012, whereas the right panel presents the relative marriage risks within each group, using 2012 as the reference category for each group (see also Table 2 in the Appendix). A uniform decline appears across individuals of all migratory backgrounds during the 2010s, with the decline accelerating during the pandemic, and recovering in 2022.

**Fig. 3:**
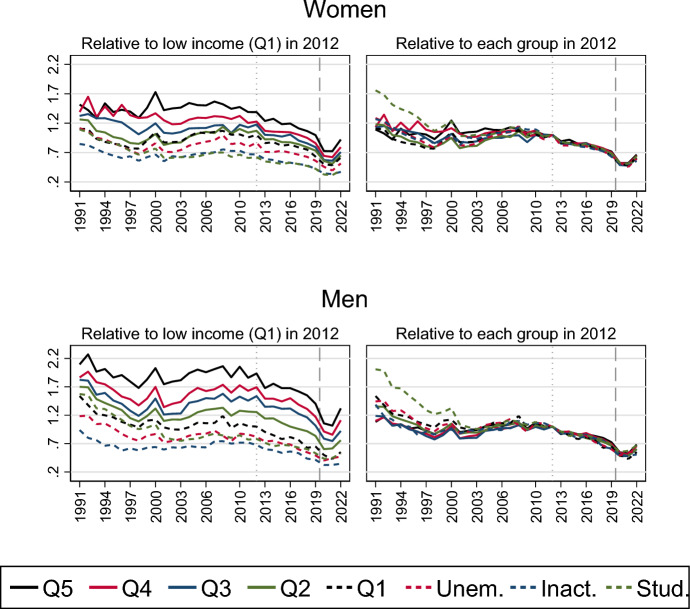
Relative risk of first marriage formation in Sweden, by calendar year and labour market activity. Piece-wise constant baseline intensity models for women and men separately. *Notes*: The left panel shows rates relative to those of low-income individuals in 2012; the right panel shows rates relative to those in 2012 for each category of labour market activity. Models control for age, parity, region of residence, parents’ country of birth and education. Q5 = Fifth quintile (high income); Q4 = Fourth quintile (medium–high income); Q3 = Third quintile (medium income); Q2 = Second quintile (medium–low income); Q1 = First quintile (low income)

A similar pattern of relative equality in marriage formation trends according to never-married women and men’s labour market status is presented in Fig. 3. The panels of the graph can be interpreted in the same way as shown in Fig. 2. Large absolute differences in marriage propensities are visible across labour market groups, with higher marriage risks the higher the earnings levels (see Table 1), but the relative decline in the 2010s occurs similarly for all groups, as well as the accelerated drop after the onset of the pandemic and the revival in 2022 (see also Table 3 in the Appendix). The 2010s marriage decline is somewhat weaker among the high-income group than among the inactive and the low-income group, as was also the case for the first-birth decline in Sweden during this decade (Ohlsson-Wijk & Andersson, 2022). For example, the marriage risk fell by 29% from 2012 to 2019 for women in the upper strata of the income distribution, whereas it fell by 37% for women in the lowest part of the income distribution. However, the marriage drop in 2020 was larger for the former than for the latter group: The marriage risk decreased by 21% from 2019 to 2020 among women with low earnings and by 27% among women with high income.

**Fig. 4:**
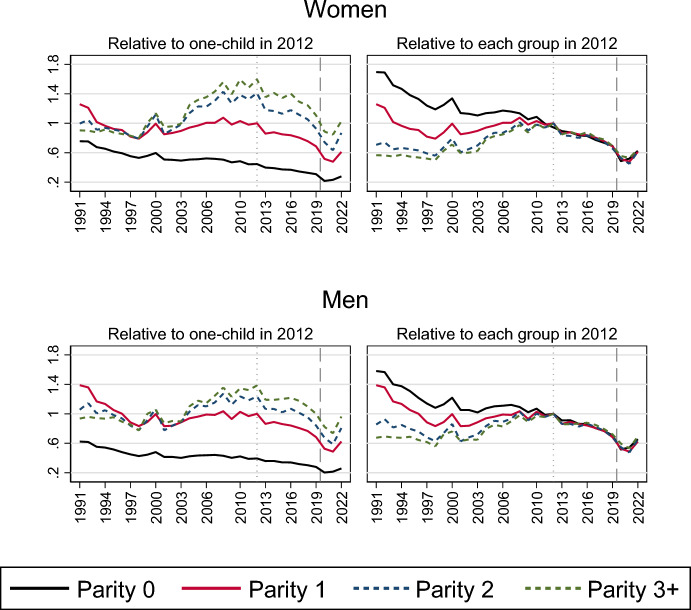
Relative risk of first marriage formation in Sweden, by calendar year and parity. Piece-wise constant baseline intensity models for women and men separately. *Notes*: The left panel shows rates relative to those of one-child mothers and fathers in 2012; the right panel shows rates relative to those in 2012 for each parity. Models control for age, region of residence, parents’ country of birth, education and labour market activity

Finally, Fig. 4—with a similar design as in Figs. 2 and 3—shows how the marriage formation trends evolved for women and men with different numbers of children. Of particular interest for our focus is that the relative marriage decline during the 2010s appeared very similar for all parity groups (see also Table 4 in the Appendix). It follows in the wake of a period of marriage increases during the first decade of the 2000s for women and men who were parents, and who continued to have higher marriage propensities than the childless during the decline period of the 2010s. For the childless, the recent decline is part of a more long-term development, with only a minor stabilization in their marriage formation levels during the early 2000s. Marriage risks decreased drastically in the first year of the Covid-19 pandemic for all parity groups, but childless women and men seem to have halted this decline in 2021. The recovery in marriage rates during 2022 was observed across all parity groups.

Our findings on trends in marriage formation thus show that the marriage risks decreased gradually during the 2010s and then sharply during the entry of the pandemic, without any notable relative differences in trend changes across social groups or parity belonging. Moreover, the observed decline does not appear to be driven by changes in the composition of the population at marriage formation ages.

### Cohabitation Trends

**Fig. 5:**
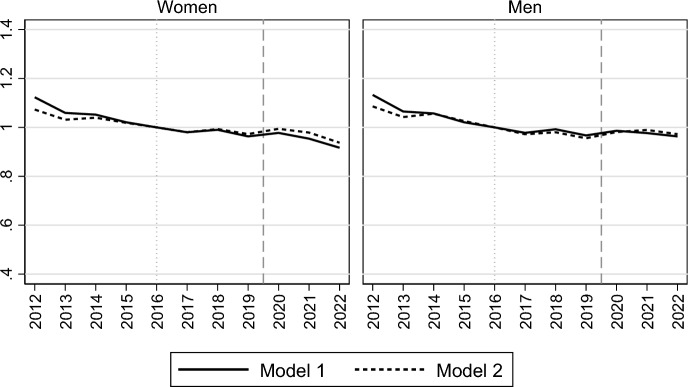
Relative risk of cohabitation formation in Sweden, by calendar year. Piece-wise constant baseline intensity models for women and men separately. *Notes*: Rates relative to rates in 2016. Model 1 controls only for age; Model 2 also controls for parity, region of residence, parents’ country of birth, education and labour market activity

We now turn to our findings regarding trends in cohabitation formation, starting with Fig. 5, which presents the main trends in the relative risks of entry into cohabitation by calendar year. In these models, we use 2016 as our baseline year for the calculation of relative risks. Results from our Model 1—which standardizes the period trends only for the age composition—show a minor decline in cohabitation formation until 2016, followed by a striking stability in union formation during the following years, including in the pandemic period. Based on our assumption that these findings reflect entry into first cohabitation, the results suggest that the decline in first cohabitation formation has been very limited.^7^

As in the case for our analyses of marriage formation, these trends seem largely unaffected by any compositional changes in terms of socio-economic status, childbearing and other measured characteristics: The results from our Model 2 in Fig. 5 are very similar to those from the simpler Model 1.

**Fig. 6:**
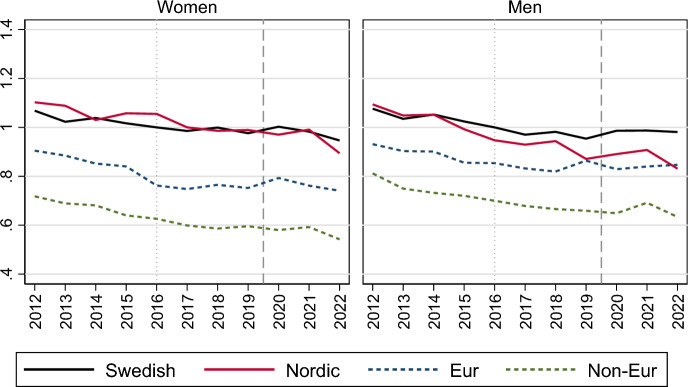
Relative risk of cohabitation formation in Sweden, by calendar year and parents’ country of birth. Piece-wise constant baseline intensity models for women and men separately. *Notes*: Rates relative to those with Swedish-born parents in 2016. Models control for age, parity, region of residence, education and labour market activity

Next, Fig. 6 shows the differences in the levels and trends in cohabitation formation according to parents’ country of birth, estimated in a model with full controls. We present these rates relative to individuals with two Swedish-born parents in 2016. First, we note that these patterns can be nicely contrasted with those for marriage formation (Fig. 2, left panel), which showed that individuals with parents born outside Europe had the highest marriage risks. In this case, men and women with Swedish- or Nordic-born parents have similar risks of cohabitation formation and those with non-Nordic European and especially non-European parents have a substantially lower propensity to enter cohabitation over the entire decade. Moreover, despite the differences in the levels of cohabitation formation across these groups, the trends show a high degree of similarity with relative stability during the latter part of the study period for most groups but with slightly declining overall trends for several groups of individuals with a migration background.

**Fig. 7:**
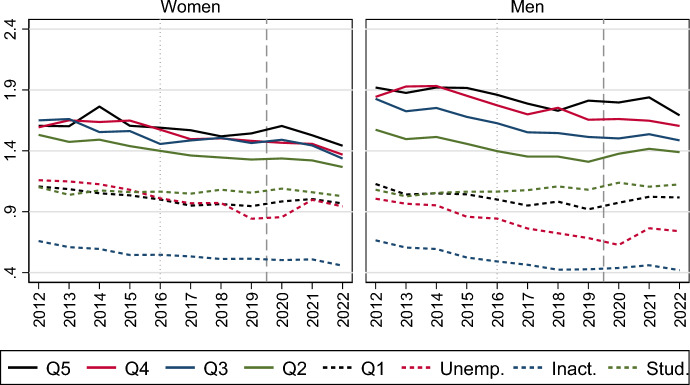
Relative risk of cohabitation formation in Sweden, by calendar year and labour market activity. Piece-wise constant baseline intensity models for women and men separately. *Notes*: Rates relative to those of low-income individuals in 2016. Models control for age, parity, region of residence, parents’ country of birth and education. Q5 = Fifth quintile (high income); Q4 = Fourth quintile (medium–high income); Q3 = Third quintile (medium income); Q2 = Second quintile (medium–low income); Q1 = First quintile (low income)

The findings on differences in levels and trends across our labour market categories are presented in Fig. 7. The findings on differences between groups in the levels of cohabitation formation correspond nicely with those observed for marriage formation (Fig. 3, left panel) and also with previous findings for first-birth risks (Ohlsson-Wijk & Andersson, 2022). Just as for marriage and becoming a parent, the high-income earners are more likely than low-income earners, unemployed and the inactive to form a cohabiting union. The only exception holds for the student group, with students having a higher cohabitation risk than the unemployed, inactive and, over most of the recent years, low-income earners. However, the trends in cohabitation formation have been strikingly similar for the different labour market groups.

**Fig. 8:**
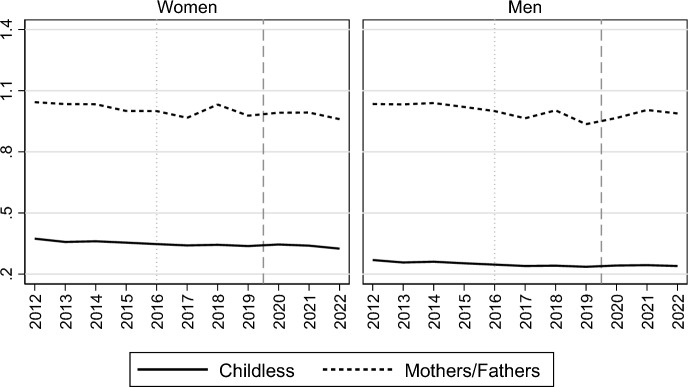
Relative risk of cohabitation formation in Sweden, by calendar year and parity. Piece-wise constant baseline intensity models for women and men separately. *Notes*: Rates relative to those of mothers and fathers in 2016. Models control for age, region of residence, parents’ country of birth, education and labour market activity

Finally, Fig. 8 shows how the relative risks of cohabitation entry have evolved for childless individuals and for individuals with children.^8^ Both childless people and parents appear to have very stable rates of cohabitation formation over the whole decade, including during the pandemic and its immediate aftermath. Cohabitation formation is mainly produced by childless people but the arrival of an out-of-union childbirth triggers much elevated levels of cohabitation formation. Our findings once more confirm that the propensities of entering a cohabiting partnership have not declined over the last decade.

## Conclusion

In this contribution, we studied recent trends in marriage and cohabitation formation in Sweden, focusing on the period during the 2010s and the subsequent Covid-19 pandemic. The 2010s was a period of declining first-birth rates, and we were interested in studying the extent to which the trends in union formation also were changing during this decade. Furthermore, we were interested in detecting whether the recent trends may have differed across different subgroups of the population, and whether any new trends were driven by changes in the composition of men and women at union formation ages. Our study reveals that Sweden experienced a slow but gradually accelerating decline in first marriage rates during the 2010s, followed by a sharp drop in marriages during the pandemic and partial recuperation thereafter. In contrast, there was very little change in the rates of cohabitation formation during the same period.

The gradual decline in first marriage rates during the 2010s shows a striking similarity with the decline previously observed for first births (Ohlsson-Wijk & Andersson, 2022), whereas the more drastic drop during the pandemic was decoupled from the corresponding development for fertility (Neyer et al., 2022). The sharp drop in marriage formation during 2020 can rather be linked to characteristics that are specific to that life-course transition, such as the practice of hosting big celebrations with family and friends (Lappegård & Noack, 2015), which was not possible during the restrictions that were applied during the course of the Covid-19 pandemic. This interpretation gains support from the subsequent resurgence in marriages during 2022, following the easing and eventual lifting of most pandemic-related restrictions in Sweden.

Moreover, we found that the decline in first marriage rates was not driven by compositional changes related to the socio-economic standing of Swedish women and men. In fact, the proportions of women and men usually more likely to enter marriage, such as those active in the labour market and with high earnings, increased during the 2010s (cf. Ohlsson-Wijk & Andersson, 2022). Without such a compositional change, there might have been an even steeper decline in first marriages. Furthermore, the falling marriage rates seemed not to be linked directly to the fertility decline either as they were not driven by changes in the parity composition among Swedish women and men during the period we study. Indeed, changes in childbearing behaviour during the 2010s, which produced increasing fractions of childless women and men, explained only a negligible portion of the first marriage fall.

Further, despite different *levels* of first marriage intensities across social groups during the entire period (e.g. transition rates being higher for individuals with high earnings), the relative decline in rates appeared strikingly homogeneous for women and men with different labour market status, migration background and other socio-demographic belonging. Indeed, the first marriage decline in the 2010s was homogeneous also for women and men with different numbers of children, even though only parents had experienced the preceding marriage uptick during the first decade of the 2000s. Marriage risks also dropped similarly for all parity groups during the pandemic.

By exploiting new register data on Swedish dwellings and households, we also analysed patterns and trends in cohabitation formation at the population level for the first time. We showed that the rates in cohabitation formation had been strikingly stable for young women and men during the period we study. They declined somewhat during the first years from 2012 onwards, stabilized after 2016 and remained rather unchanged even during the course of the pandemic.

As for our findings for marriage formation, the trends in cohabitation formation were largely unaffected by compositional changes in the population across different socio-demographic groups. Moreover, by differentiating trends according to labour market status, migration background and parental status, no substantial differences in trend developments appeared across groups over the 2010s or during the Covid-19 period.

As our findings demonstrate declining marriage formation trends but relatively stable cohabitation formation trends in the last decade, we might conclude that the fertility decline that occurred in Sweden in the 2010s has not been produced by a decline in the formation of new cohabiting partnerships. This aligns with previous studies on Sweden and other Nordic countries, showing that the recent fertility fall was primarily driven by a declining propensity among childless cohabiting people to become parents (Neyer et al., 2022; for Finland, see Hellstrand et al., 2022). The fertility decline appears to be a distinct development among childless cohabiting women and men (considering that Swedes rarely marry or have children without cohabiting first (Andersson & Philipov, 2002; Andersson et al., 2017)). Added to this picture might be an equally distinct development among cohabiting people to become less prone to change their union status to that of a marital union.

The findings of our study, coupled with that of previous findings of declining first-birth rates, suggest that young Swedish adults are becoming increasingly hesitant to take the next steps in terms of further family commitments, both in terms of marriage and childbearing. This tendency fits with arguments that a process of marketization of personal relationships has made people less inclined to establish firm commitments (Illouz, 2019). In Sweden, as in other modern societies new romantic relationships are increasingly often established by means of internet dating tools (Bergström, 2019). This change in dating behaviour, however, has not led to a decline in the formation of new cohabiting unions. As we have shown, young Swedes still enter cohabiting unions with the same intensity as before, but apparently have become less inclined to transition their relationship status by forming a marriage or becoming parents.

To sum up, the Swedish fertility decline during the 2010s was joined by a parallel trend of declining marriage rates, but not of less cohabitations. The trend of declining marriage rates was an independent development and not produced by changes in the parity composition of non-married people. However, marriage decisions are still strongly linked to childbearing decisions (Holland, 2013; Lappegård & Noack, 2015) and it is plausible that some of the factors that may have driven the fertility change have also contributed to the marriage decline. These factors are by no means verified but increases in uncertainties about the future and perceived uncertainties in people’s lives resulting from globalization dynamics, changing labour market structures, new information technologies, new modes of forming personal relationships and related political and cultural polarization, may thus negatively affect not only fertility decisions (Neyer et al., 2022; Vignoli et al., 2020) but also marriage intentions. In contrast, forming a cohabitation union and remaining in a cohabiting state may be better compatible with a situation of perceived uncertainties, as cohabitations require less commitment than that required by marriage or parenthood (Guetto et al., 2021).

Our study made several contributions. Besides analysing cohabitation trends in the Swedish population for the first time, it provided new evidence on first marriage trends, and new insight into the relationship between union dynamics and fertility development. However, it also poses new questions for future research. A focus on patterns and trends in the dissolution of cohabiting unions would produce more in-depth information on the changing dynamics in such unions (Jalovaara & Andersson, 2023). The state of being in a childless cohabiting union is normally a quite unstable and transient one (Andersson et al., 2017). However, if union dissolution rates have not increased during the period we study, it may well be that this state has become a new and more prominent family form in Swedish family dynamics. A more in-depth focus on the competing risks of leaving the status of being a childless cohabitee would add further insight into recent family change. In this respect, future research could investigate which factors matter for couples in their decisions or possibilities to go forward with marriage and/or parenthood, compared to staying at *status quo* or dissolving their union.

## Figures and Tables

**Fig. 9:**
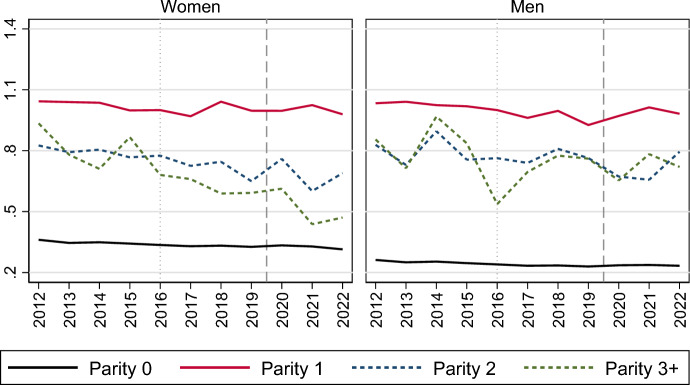
Relative risk of cohabitation formation in Sweden, by calendar year and parity. Piece-wise constant baseline intensity models for women and men separately. *Notes*: Rates relative to those of one-child mothers and fathers in 2016. Models control for age, region of residence, parents’ country of birth, education and labour market activity

## Data Availability

The original individual-level data are stored at Statistics Sweden and available only to approved researchers.

## Appendix

See Tables 2, 3, 4 and Fig. 9.

**Table 2:** Relative risk of first marriage formation in Sweden during the 2010s and the Covid-19 pandemic, by parents’ country of birth. Piece-wise constant baseline intensity models for women and men separately, presented in Fig. 2

	Swedish	Nordic	European	Non-European
*Women*				
2010	1.04	0.98	1.03	1.10
2011	0.97	0.96	0.92	1.00
2012	1	1	1	1
2013	0.87	0.85	0.84	0.88
2014	0.86	0.85	0.84	0.87
2015	0.83	0.82	0.85	0.83
2016	0.83	0.84	0.85	0.81
2017	0.79	0.81	0.80	0.72
2018	0.74	0.75	0.77	0.69
2019	0.68	0.64	0.72	0.65
2020	0.51	0.53	0.55	0.51
2021	0.48	0.48	0.58	0.56
2022	0.63	0.61	0.62	0.56
*Men*				
2010	1.04	1.03	1.04	1.05
2011	0.97	0.99	1.00	1.00
2012	1	1	1	1
2013	0.88	0.87	0.90	0.98
2014	0.88	0.90	0.93	0.97
2015	0.85	0.83	0.87	0.90
2016	0.86	0.85	0.92	0.89
2017	0.81	0.79	0.86	0.83
2018	0.76	0.80	0.83	0.77
2019	0.69	0.64	0.74	0.72
2020	0.53	0.54	0.57	0.58
2021	0.50	0.49	0.57	0.63
2022	0.65	0.64	0.72	0.68

The table shows rates relative to those in 2012 for each category of parents’ birth country

**Table 3:** Relative risk of first marriage formation in Sweden during the 2010s and the Covid-19 pandemic, by labour market activity. Piece-wise constant baseline intensity models for women and men separately, presented in Fig. 3

	Inactive	Student	Unempl	Low inc. (Q1)	M-L inc. (Q2)	Med. inc. (Q3)	M-H inc. (Q4)	High inc. (Q5)
*Women*								
2010	1.09	1.01	1.03	1.00	1.08	1.06	1.07	1.00
2011	1.00	0.96	0.97	0.96	0.98	1.00	0.98	0.92
2012	1	1	1	1	1	1	1	1
2013	0.88	0.86	0.86	0.85	0.87	0.89	0.88	0.82
2014	0.86	0.88	0.87	0.85	0.86	0.92	0.86	0.82
2015	0.81	0.82	0.82	0.83	0.86	0.85	0.81	0.84
2016	0.79	0.84	0.82	0.85	0.85	0.86	0.85	0.80
2017	0.75	0.79	0.79	0.77	0.82	0.80	0.81	0.77
2018	0.72	0.75	0.74	0.73	0.77	0.77	0.76	0.72
2019	0.63	0.63	0.68	0.67	0.70	0.72	0.68	0.65
2020	0.52	0.50	0.52	0.49	0.52	0.52	0.52	0.53
2021	0.50	0.48	0.48	0.48	0.51	0.52	0.51	0.46
2022	0.56	0.61	0.61	0.60	0.65	0.66	0.64	0.59
*Men*								
2010	1.06	1.06	1.02	0.99	1.02	1.06	1.08	1.07
2011	1.06	1.01	1.01	0.95	0.96	0.96	0.98	1.05
2012	1	1	1	1	1	1	1	1
2013	0.89	0.91	0.91	0.88	0.88	0.87	0.91	0.90
2014	0.87	0.85	0.91	0.88	0.88	0.91	0.83	0.92
2015	0.79	0.78	0.85	0.86	0.85	0.87	0.87	0.86
2016	0.83	0.81	0.80	0.86	0.89	0.87	0.85	0.84
2017	0.79	0.76	0.76	0.80	0.82	0.84	0.80	0.73
2018	0.64	0.63	0.73	0.76	0.77	0.80	0.77	0.67
2019	0.61	0.65	0.67	0.66	0.69	0.72	0.68	0.60
2020	0.48	0.50	0.48	0.51	0.54	0.55	0.55	0.49
2021	0.48	0.43	0.49	0.49	0.51	0.53	0.57	0.54
2022	0.51	0.55	0.60	0.60	0.66	0.68	0.67	0.57

The table shows rates relative to those in 2012 for each category of labour market activity. Q1 = First quintile; Q2 = Second quintile; Q3 = Third quintile; Q4 = Fourth quintile; Q5 = Fifth quintile

**Table 4:** Relative risk of first marriage formation in Sweden during the 2010s and the Covid-19 pandemic, by parity. Piece-wise constant baseline intensity models for women and men separately, presented in Fig. 4

	Parity 0	Parity 1	Parity 2	Parity 3+
*Women*				
2010	1.09	1.03	0.98	1.00
2011	0.99	0.98	0.94	0.93
2012	1	1	1	1
2013	0.89	0.86	0.84	0.85
2014	0.88	0.88	0.83	0.88
2015	0.84	0.85	0.80	0.84
2016	0.83	0.84	0.84	0.88
2017	0.77	0.80	0.79	0.81
2018	0.73	0.76	0.75	0.78
2019	0.69	0.69	0.66	0.69
2020	0.49	0.52	0.53	0.56
2021	0.52	0.48	0.45	0.53
2022	0.62	0.61	0.62	0.64
*Men*				
2010	1.07	1.03	1.00	0.97
2011	0.99	0.97	0.96	0.96
2012	1	1	1	1
2013	0.91	0.86	0.86	0.86
2014	0.91	0.89	0.86	0.86
2015	0.86	0.86	0.83	0.87
2016	0.86	0.84	0.87	0.88
2017	0.81	0.81	0.82	0.85
2018	0.77	0.77	0.77	0.79
2019	0.71	0.68	0.68	0.72
2020	0.52	0.53	0.55	0.59
2021	0.54	0.49	0.48	0.53
2022	0.66	0.62	0.65	0.70

The table shows rates relative to those in 2012 for each parity

## Footnotes

1 This follows from changes in the registration of legal residence so that each individual is to be registered as living in a unique apartment rather than just a property that in many cases contains several apartments. For this purpose, a new dwelling register was created and the dwelling and population registers were linked to each other. With this change in practice, Sweden was able to produce proper household data and switch to a system with fully register-based censuses (Andersson et al., 2023).

2 Compared to the marriage analysis, we opted to focus on a younger sample also because entry into first cohabitation typically occurs at much earlier ages than entry into first marriage (see Sect. 2.2).

3 Nordic-born parents refer to those born in Denmark, Norway, Finland or Iceland. Non-Nordic European-born parents include those born in any European country, excluding Sweden or other Nordic nations.

4 This lower level refers to a previous value of an administrative measure used to establish cut-off points for public transfers in Sweden and represents an income level that is far below what can sustain a basic livelihood.

5 Due to the accuracy of annual measurement when analysing entry into cohabitation, it is possible to miss short cohabitation spells lasting less than one year. For instance, cohabitation episodes that start in the same year as a marriage are not traced in our data.

6 The relative risk of marriage formation in 2020 compared to 2019 is equal to 0.75 (0.72/0.54) for women and 0.76 (0.73/0.56) for men, corresponding to the abovementioned decline of 24–25% in one year.

7 As a robustness check, we also restricted the population under risk to childless individuals in 2011, thus reducing any incidence of previous cohabitation. Further, we re-ran our analyses based exclusively on the cohorts born in 1994 or later, who all turned 18 during our study period and for which we can always study the early life-course segment of interest. These sensitivity analyses confirm the results presented here.

8 For a more detailed presentation of differences across parity groups, see Fig. 9 in the Appendix.
